# Chinese herbal formulas for Hashimoto’s thyroiditis based on the thyroid-gut axis: multitarget synergistic mechanisms and boundaries of evidence

**DOI:** 10.3389/fendo.2026.1840643

**Published:** 2026-06-18

**Authors:** Yujia Wu, Hongquan Shen, Jie Wang, Wenjun Sha, Zhidan Zhang

**Affiliations:** Putuo Hospital, Shanghai University of Traditional Chinese Medicine, Shanghai, China

**Keywords:** Chinese herbal formulas, gut microbiota, Hashimoto’s thyroiditis, immune homeostasis, intestinal barrier, thyroid-gut axis

## Abstract

**Background:**

Hashimoto’s thyroiditis (HT) is a common autoimmune thyroid disease. Although levothyroxine replacement therapy can correct hypothyroidism, it does not directly reverse the autoimmune process. The thyroid-gut axis provides a mechanistic framework for understanding how intestinal barrier injury, microbial dysbiosis, disrupted immune homeostasis, and endocrine disturbance jointly contribute to HT.

**Objective:**

This review systematically summarizes current evidence on Chinese herbal formulas that intervene in HT through the thyroid-gut axis and proposes a target-combination-based mechanistic framework.

**Methods:**

Original studies published from 2015 to 2025 were searched in CNKI, PubMed, Embase, and the Cochrane Library. Thirty-three records were initially identified, and eight studies on eligible Chinese herbal formulas were ultimately included.

**Results:**

The included formulas covered four pathological nodes: intestinal barrier repair (A), gut microbiota remodeling (B), immune homeostasis reconstruction (C), and endocrine function restoration (D). The observed target combinations included A+C, B+C, B+C+D, and A+B+C+D. On this basis, we propose a four-level linkage model to integrate the available evidence.

**Conclusion:**

Chinese herbal formulas may modulate HT pathology through multitarget regulation of the thyroid-gut axis. However, the current evidence is mainly derived from heterogeneous preclinical animal studies and remains largely correlative. Future studies should include randomized controlled clinical trials, causal microbiome experiments, standardized outcome assessment, safety evaluation, and pharmacokinetic investigation.

## 1 Introduction

Hashimoto’s thyroiditis (HT) is a common chronic autoimmune thyroid disease characterized by lymphocytic infiltration of the thyroid, follicular destruction, persistent elevation of thyroid autoantibodies, and gradual progression to hypothyroidism in some patients (1, 2). Current clinical management still relies primarily on levothyroxine replacement to correct hypothyroidism, but this approach does not directly interrupt the autoimmune process (2). The development of HT involves complex interactions among genetic susceptibility, immune dysregulation, environmental exposure, and metabolic factors (3). In recent years, the thyroid-gut axis has emerged as an important perspective for explaining the pathogenesis of HT and identifying potential therapeutic targets. This axis involves the intestinal barrier, gut microbiota, immune tolerance, endocrine signaling, and energy metabolism (4, 5).

Chinese herbal formulas are characterized by multicomponent, multistep, and holistic regulation within traditional Chinese medicine. This feature makes them suitable for explaining interventions in HT, a network-driven autoimmune disease. In this review, we systematically retrieved and summarized original studies and developed a four-level linkage model based on four pathological nodes: intestinal barrier injury (A), gut microbiota dysbiosis (B), immune homeostasis imbalance (C), and endocrine homeostasis disturbance (D). It should be emphasized that this model is a conceptual framework derived from current evidence and should not be regarded as a rigorously proven causal chain.

## 2 Autoimmune basis of HT and the pathological rationale for the thyroid-gut axis

### 2.1 Autoimmune mechanisms of HT

Autoimmune injury in HT involves both cellular and humoral immunity. Polymorphisms in HLA class II genes and immune regulatory genes, including CTLA-4, PTPN22, and FOXP3, can increase susceptibility to autoimmune thyroid disease (6, 7). In an inflammatory microenvironment, thyroid follicular epithelial cells may aberrantly express HLA class II molecules and present autoantigens such as thyroid peroxidase (TPO) to CD4+ T cells (8). This process mediates pathogenic Th1 and Th17 immune responses, triggers inflammatory cascades, disrupts the Th17/Treg immune axis, and promotes the onset and progression of HT (9, 10). Meanwhile, activated B cells produce TPOAb and TgAb, further contributing to thyroid follicular destruction (11). Therefore, HT is not caused by a single molecular abnormality, but by a chronic inflammatory network consisting of antigen presentation, proinflammatory T-cell polarization, autoantibody production, and impaired immune tolerance.

### 2.2 Four bidirectional pathological nodes of the thyroid-gut axis

The thyroid-gut axis is not a one-way pathway in which the gut simply affects the thyroid. Instead, it is a bidirectional feedback network. Gut abnormalities may amplify autoimmune responses in the thyroid, whereas thyroid dysfunction may in turn affect the intestinal barrier, gut microbiota, and immune microenvironment. Based on current evidence, this review summarizes the axis into four interrelated pathological nodes.

Intestinal barrier injury (A). In the gut-to-thyroid direction, serum zonulin is a key regulator of intestinal tight junctions. It can induce dissociation of tight-junction protein complexes and increase intestinal permeability (12, 13). Increased permeability facilitates the entry of microbial products such as lipopolysaccharide (LPS) into the circulation, activates thyroid-localized inflammation through pathways such as TLR4/NF-kappaB, and disrupts Th17/Treg immune balance (14, 15). In the thyroid-to-gut direction, hypothyroidism is associated with altered intestinal permeability and elevated serum LPS, suggesting that thyroid hormone deficiency may weaken intestinal barrier function (16).

Gut microbiota dysbiosis (B). In the gut-to-thyroid direction, gut microbiota-derived metabolites, such as short-chain fatty acids, secondary bile acids, and tryptophan derivatives, can aggravate autoimmune thyroid injury by disturbing the Treg/Th17 balance (17). Clinical studies and meta-analyses suggest that patients with HT show changes in gut microbial diversity and increased abundance of certain genera, some of which are positively correlated with TPOAb (18). In the thyroid-to-gut direction, hypothyroidism may also reshape the gut microbiota by slowing intestinal motility, increasing proinflammatory bacteria, and inhibiting the proliferation of beneficial bacteria (16).

Immune homeostasis imbalance (C). In the gut-to-thyroid direction, reduced short-chain fatty acids impair Treg differentiation, while LPS translocation activates inflammatory cascades. Together, these events aggravate Th17/Treg imbalance, promote the release of proinflammatory cytokines, induce ferroptosis in thyroid follicular epithelial cells, and ultimately exacerbate structural thyroid injury (19–22). In the thyroid-to-gut direction, thyroid hormone deficiency caused by HT may impair intestinal epithelial barrier function through TRalpha1 signaling (23). Proinflammatory cytokines originating from the thyroid, including IL-17A and IFN-gamma, may reach intestinal tissues through the bloodstream, disrupt local immune tolerance, and induce secondary inflammation (9).

Endocrine homeostasis disturbance (D). In the gut-to-thyroid direction, the gut microbiota participates in the enterohepatic circulation of thyroid hormones and promotes thyroid hormone reabsorption (24). In HT, the abundance of proinflammatory bacterial genera is negatively correlated with FT3 levels (25), indicating that the gut microbiota may influence thyroid hormone bioavailability. In the thyroid-to-gut direction, thyroid hormones regulate intestinal epithelial proliferation and differentiation through TRalpha1, and thyroid hormone dysfunction can lead to impaired intestinal motility (24). In hypothyroidism, bile acid metabolism is downregulated, and TSH is negatively correlated with total bile acids (26, 27), suggesting that hormonal deficiency may further worsen the intestinal environment. Levothyroxine replacement therapy can modulate gut microbial structure (28). Thus, endocrine disturbance is both a consequence of thyroid injury and a potential factor that maintains gut dysbiosis and barrier injury.

### 2.3 Rationale for multitarget intervention by Chinese herbal formulas

The four nodes described above jointly form a self-reinforcing pathological loop in the thyroid-gut axis of HT. Autoimmune thyroid injury and endocrine disturbance can aggravate intestinal barrier damage and microbial dysbiosis, while gut abnormalities further amplify thyroid inflammation through LPS translocation, disordered metabolites, and reduced immune tolerance. Chinese herbal formulas contain multiple chemical constituents and may theoretically act on the barrier, microbiota, immune, and endocrine levels simultaneously. Therefore, they may better fit the network-regulatory characteristics of HT than single-target agents. Nevertheless, multitarget synergy should not be interpreted as a pharmacological synergistic effect that has already been proven. Current evidence mainly indicates that different formulas cover different target combinations, and their true synergistic relationships still require validation through causal experiments and clinical trials.

## 3 Literature search and study selection

This review followed the PRISMA 2020 statement for literature retrieval and screening. The databases searched included CNKI, PubMed, Embase, and the Cochrane Library. The search covered studies published from January 1, 2015 to December 31, 2025. Search terms included disease-related terms (Hashimoto’s thyroiditis, autoimmune thyroiditis, experimental autoimmune thyroiditis, and their Chinese equivalents), gut-axis-related terms (gut microbiota, gastrointestinal microbiome, intestinal mucosa, intestinal barrier, thyroid-gut axis, and their Chinese equivalents), and intervention-related terms (traditional Chinese medicine, Chinese herbal formula, decoction, granule, tablet, and their Chinese equivalents).

The inclusion criteria were as follows: (1) original studies; (2) studies involving HT, autoimmune thyroiditis, or experimental autoimmune thyroiditis (EAT) animal models; (3) interventions using Chinese herbal formulas rather than isolated compounds, Western medicines, probiotics, acupuncture alone, or non-Chinese-medicine interventions; and (4) reporting at least one type of outcome related to gut microbiota, intestinal barrier, thyroid hormones, or thyroid autoantibodies. Exclusion criteria were reviews, systematic reviews, meta-analyses, comments, editorials, case reports, popular science articles, duplicate publications, unavailable full texts, incomplete data, disease models inconsistent with HT/EAT, or studies without thyroid-gut-axis-related outcomes.

**Figure 1:**
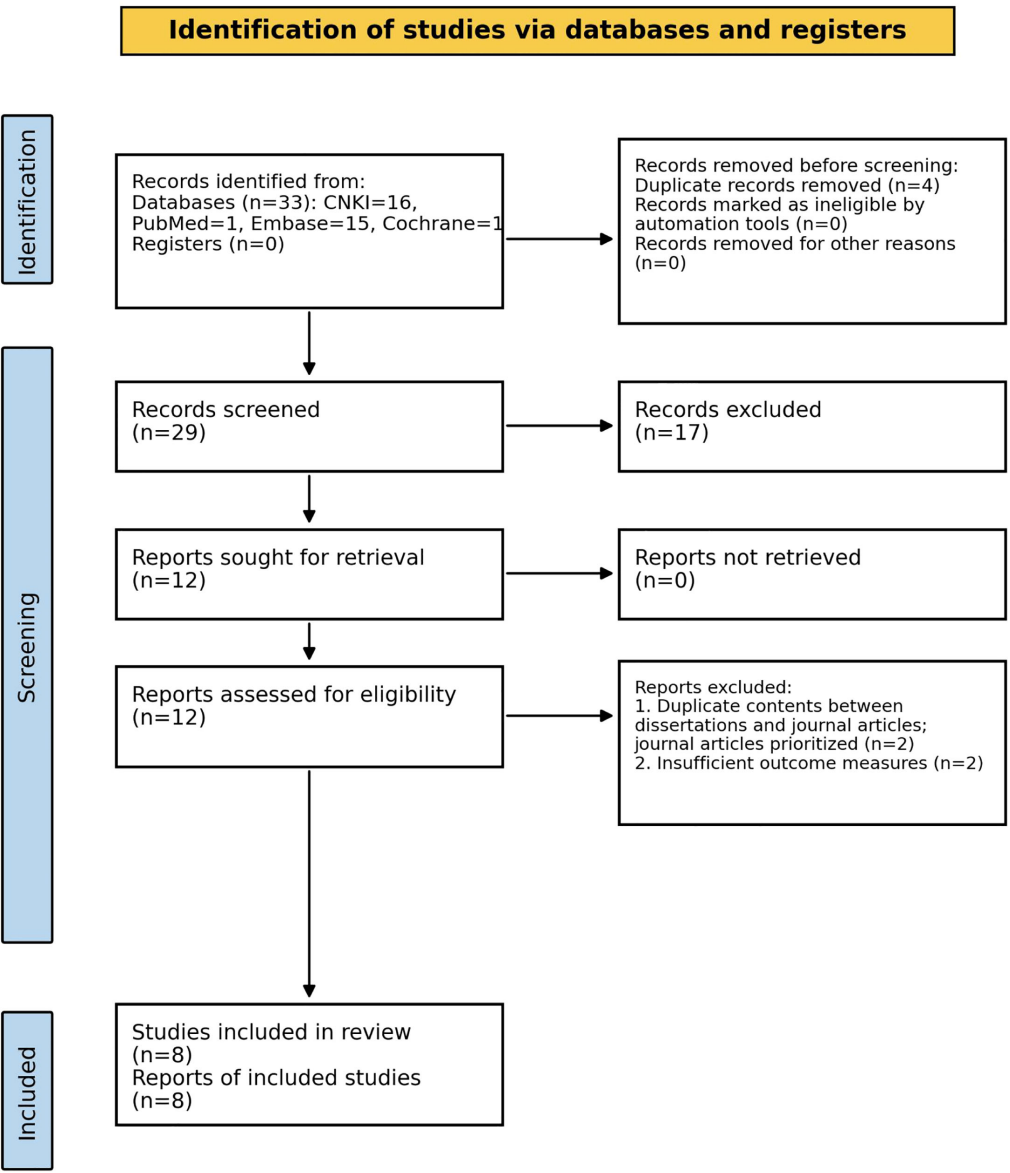
PRISMA flow diagram of literature retrieval and study selection.

A total of 33 records were initially identified. After removal of four duplicates, 29 records entered title and abstract screening, of which 17 were excluded. Twelve full-text reports were then retrieved and assessed for eligibility. Four reports were further excluded: two because journal articles and dissertations had duplicate content and the journal articles were prioritized, and two because the outcome measures were insufficient. Finally, eight studies were included. Because the included studies differed markedly in formula composition, animal models, intervention duration, and outcome measures, meta-analysis was not performed. Instead, a narrative evidence map was used to extract formula names, model types, target coverage, main outcomes, and evidence limitations. The study selection process is shown in **Figure 1**.

## 4 Evidence map of Chinese herbal formulas modulating the thyroid-gut axis

The eight included Chinese herbal formulas showed different patterns of target coverage (**Table 1**). Two formulas mainly covered intestinal barrier and immune targets (A+C), two mainly covered microbiota and immune targets (B+C), three covered microbiota, immune, and endocrine targets (B+C+D), and one covered all four targets (A+B+C+D). This gradient of target coverage suggests that formulas may have hierarchical regulatory features. However, each formula is currently supported by only one original study and should therefore be regarded as preliminary evidence rather than a definitive conclusion.

**Table 1:** Evidence map of Chinese herbal formulas regulating the thyroid-gut axis in HT/EAT. .

Formula	Targets	Proposed mechanism	Model	HT-related outcomes	Gut-axis-related outcomes	Evidence limitations	Ref.
Kangjiafang Granules	B+C+D	Regulated CD4+ T cells (reduced Th1 and increased Treg) and inflammatory cytokines.	Active-immunization-induced EAT (female Lewis rats).	Reduced TPOAb and TgAb; improved T3/T4/FT3/FT4 and thyroid pathology; regulated Th1/Th2 and Th17/Treg balance.	Reduced microbial diversity; increased F/B ratio, Faecalibacterium, and Bifidobacterium; decreased Escherichia-Shigella.	Animal experiment; single study; requires replication.	(29)
Xiaoyao Bushen Formula	B+C+D	Regulated oxidative stress and gut microbiota.	Active immunization plus high-iodine-induced EAT (male SD rats).	Reduced TPOAb and TgAb; increased TSH; reduced MDA and ROS; increased SOD and GPx; improved pathology.	Decreased Firmicutes, F/B ratio, and Enterobacteriaceae; increased Bacteroidetes, S24-7, and Allobaculum.	Animal experiment; mechanism remains mainly correlative.	(30)
Yiqi Huatan Huoxue Formula	B+C	Altered gut microbial structure.	Active-immunization-induced AIT (female CBA/J mice).	Alleviated thyroid lymphocytic infiltration and reduced TgAb.	Reduced Firmicutes and F/B ratio; increased Bacteroidetes and Actinobacteria.	Animal experiment; did not cover barrier or endocrine indicators.	(31)
Jieyu Zichong Granules	B+C+D	Regulated gut microbiota and indirectly affected immune homeostasis.	Active immunization plus high-iodine-induced EAT with ovarian reserve decline (female Kunming mice).	Improved thyroid pathological score; reduced TgAb and TPOAb; improved ovarian reserve.	Increased Lactobacillales, Lactobacillaceae, Lactobacillus, and L. johnsonii; decreased Enterobacteriaceae and Escherichia.	Animal experiment; extrapolation from a composite disease model is limited.	(32)
Buzhong Yiqi Granules	A+C	Improved small-intestinal mucosal ultrastructure.	Active immunization plus high-iodine-induced EAT (female SD rats).	Reduced TPOAb and TgAb; improved thyroid pathology.	Reduced zonulin; improved microvilli, tight junctions, and mitochondrial ultrastructure.	Animal experiment; overall gut microbiota changes were not assessed.	(33)
Buzhong Yiqi Decoction	A+C	Upregulated tight-junction proteins and improved the intestinal mucosal barrier.	High-iodine-induced AIT (NOD.H-2h4 mice).	Reduced TgAb and improved thyroid pathology.	Upregulated ZO-1, claudin-1, and occludin; reduced serum and intestinal-content LPS.	Dissertation; relatively limited level of evidence.	(34)
Ruanjian Xiaoying Granules	B+C+D	Regulated metabolomic profiles and gut microbiota.	EAT model combined with liver-qi stagnation and spleen-deficiency syndrome (female SD rats).	Reduced TgAb and TPOAb; increased TSH; improved general condition.	Decreased Firmicutes and Prevotella; increased Proteobacteria and Escherichia.	Dissertation; requires independent replication and mechanistic validation.	(35)
Qijian Xiaoying Formula	A+B+C+D	Regulated microbiota and increased tight-junction proteins and sIgA.	Active immunization plus high-iodine-induced AIT (female SD rats).	Reduced FT3, FT4, TgAb, TPOAb, and IFN-gamma; increased IL-10; improved pathology.	Reduced microbial diversity, Firmicutes, and F/B ratio; increased Bacteroidetes and Lactobacillus; increased ZO-1, occludin, and sIgA.	Animal experiment; the only study covering all four targets, but still based on single-study evidence.	(36)

A, intestinal barrier repair; B, gut microbiota remodeling; C, immune homeostasis reconstruction; D, endocrine function restoration.

Each formula in this table is currently supported by only one original study. These results should be interpreted as preliminary, hypothesis-generating evidence rather than definitive evidence of clinical efficacy.

### 4.1 Target-combination patterns and the four-level linkage model

**Figure 2:**
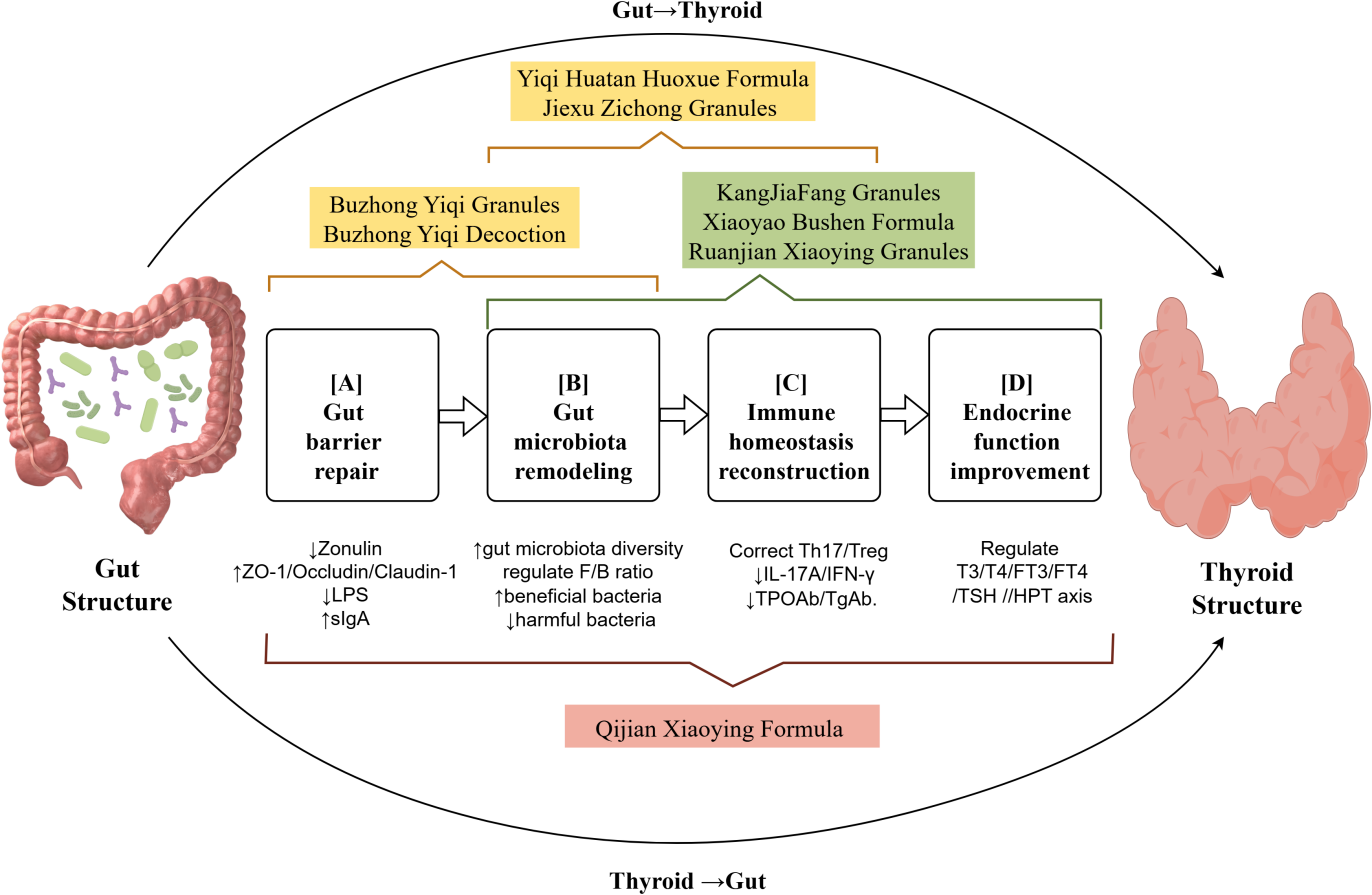
Multitarget cascade model by which Chinese herbal formulas may intervene in HT through the thyroid-gut axis. The model includes four nodes: intestinal barrier repair **(A)**, gut microbiota remodeling **(B)**, immune homeostasis reconstruction **(C)**, and endocrine function restoration **(D)**, and shows the target coverage of different formulas. This model is a hypothesis-generating framework and requires validation through causal experiments and clinical studies.

Based on the included studies, we propose a four-level linkage model: intestinal barrier repair (A) -> gut microbiota remodeling (B) -> immune homeostasis reconstruction (C) -> endocrine function improvement (D). This sequence does not imply that all formulas follow a fixed linear mechanism. Rather, it provides an explanatory framework for understanding how improvement in upstream gut abnormalities may create conditions for downstream immune and endocrine recovery, while improved thyroid function may in turn stabilize the intestinal environment (**Figure 2**).

A+C formulas. Buzhong Yiqi Granules and Buzhong Yiqi Decoction mainly covered intestinal barrier and immune outcomes. The former reduced zonulin and improved small-intestinal mucosal ultrastructure, whereas the latter upregulated ZO-1, claudin-1, and occludin and reduced LPS (33, 34). Both studies observed improvement in thyroid antibodies or pathological injury, suggesting that barrier repair may indirectly alleviate thyroid immune responses by reducing inflammatory stimulation. However, because these two studies did not systematically assess overall gut microbiota changes, the role of microbiota remodeling remains unclear.

B+C formulas. Yiqi Huatan Huoxue Formula and Jieyu Zichong Granules mainly covered microbiota and immune outcomes (31, 32). Both studies reported changes in gut microbial structure accompanied by improvement in antibodies or thyroid inflammation, suggesting that microbiota remodeling may participate in the restoration of immune tolerance. Nevertheless, these studies cannot determine whether microbiota changes are a cause of therapeutic efficacy, a consequence of treatment, or a phenomenon occurring in parallel with other pharmacological processes.

B+C+D formulas. Kangjiafang Granules, Xiaoyao Bushen Formula, and Ruanjian Xiaoying Granules covered microbiota-, immune-, and endocrine-related outcomes (29, 30, 35). Kangjiafang Granules regulated CD4+ T-cell subsets and microbial structure; Xiaoyao Bushen Formula affected oxidative stress and gut microbiota; and Ruanjian Xiaoying Granules involved metabolomic and microbial changes. This group of studies suggests possible cross-system regulation, but it has not yet been proven that microbiota changes directly mediate endocrine functional improvement.

A+B+C+D formula. Qijian Xiaoying Formula is currently the only formula that has reported outcomes covering all four nodes (36). The study showed that this formula increased ZO-1, occludin, and sIgA, regulated gut microbial structure, reduced inflammatory cytokines and autoantibodies, and affected thyroid-hormone-related indicators. Because it covers the complete pathway, Qijian Xiaoying Formula may serve as an important candidate formula for future mechanistic validation. However, current evidence still comes from a single animal study and requires independent replication.

## 5 Discussion and perspectives

### 5.1 Mechanistic integration and clinical translational potential

This review proposes a mechanistic framework centered on target combinations and pathological cascades. The four-level linkage model moves beyond the general description of ‘multiple targets and multiple pathways’ and organizes the available evidence around the bidirectional pathological loop of the thyroid-gut axis. The model emphasizes that the effects of Chinese herbal formulas should not be understood only as a one-way pathway from formula to gut, immune regulation, and thyroid improvement. Instead, they should be interpreted within the mutually reinforcing cycle between the gut-to-thyroid and thyroid-to-gut directions.

From a clinical perspective, if future studies confirm that patients with HT can be stratified into subtypes dominated by barrier injury, microbial dysbiosis, immune inflammation, or endocrine disturbance, formula selection may gradually shift from experience-based treatment to individualized regulation based on target combinations. However, this concept remains a research direction. Current evidence is insufficient to support clinical recommendations or guideline-level conclusions.

### 5.2 Current evidence boundaries and major limitations

First, the available evidence is entirely derived from preclinical animal studies. No randomized controlled clinical trial has confirmed that Chinese herbal formulas can treat HT by regulating the thyroid-gut axis. Active-immunization-induced EAT, high-iodine-induced AIT, and disease-syndrome combined models can simulate some immunopathological features of human HT, but they cannot fully reproduce human genetic susceptibility, long-term chronic progression, dietary environment, concomitant medication use, and interindividual microbiome variability.

Second, target detection is incomplete. Except for Qijian Xiaoying Formula, most studies did not simultaneously assess the intestinal barrier, gut microbial structure, immune phenotyping, thyroid autoantibodies, and thyroid hormones. Therefore, the A -> B -> C -> D cascade model remains an inference based on correlative results and cannot yet prove that improvement in upstream targets leads to restoration of downstream targets. Future studies should use time-series sampling, mediation analysis, germ-free animals, fecal microbiota transplantation, and pathway-blocking experiments to verify the causal chain.

Third, each formula is currently supported by only one study, and some evidence comes from dissertations. Replication is insufficient. The included studies differ markedly in animal strain, model construction, intervention duration, sequencing strategy, taxonomic resolution, and outcome measures, which limits evidence comparability and pooling.

Fourth, safety and drug-interaction studies are insufficient. Patients with HT commonly use levothyroxine and may also take selenium supplements, iodine-containing supplements, lipid-lowering agents, or hypoglycemic drugs. Whether Chinese herbal formulas affect levothyroxine absorption, liver and kidney function, coagulation, gastrointestinal tolerance, and long-term safety remains insufficiently evaluated. Without these data, the clinical translation of formulas targeting the thyroid-gut axis lacks a clear safety boundary.

Fifth, the oral bioavailability and intestinal stability of active constituents require further study. Flavonoid glycosides, saponins, polysaccharides, alkaloids, and other constituents in Chinese herbal formulas differ substantially in solubility, gastrointestinal degradation, absorption, microbial transformation, and intestinal retention. For example, apigenin glycosides may undergo hydrolysis or enzymatic degradation in the gastrointestinal tract and may not reach target sites in the parent form. Future studies may consider intestinal-targeted delivery, protective carriers, prodrug design, and microbiota-responsive release systems to improve local exposure and reproducibility of pharmacological effects.

### 5.3 Future research directions

Future research can be advanced at three levels. First, rigorously designed prospective, randomized, double-blind, placebo-controlled clinical trials should be conducted in patients with clearly diagnosed HT. These trials should simultaneously monitor TPOAb, TgAb, thyroid hormones, gut metagenomes, zonulin, LPS, sIgA, and safety indicators. The intervention period should preferably last at least 12 weeks and include follow-up after treatment withdrawal to evaluate durability and safety.

Second, causal mechanisms should be validated using germ-free animals, antibiotic-mediated microbiota depletion, fecal microbiota transplantation, and gene knockout approaches. For example, transplanting fecal microbiota from formula-treated EAT animals into untreated EAT recipient animals could test whether microbiota remodeling is sufficient to transmit therapeutic effects. Blocking TLR4, GPR41/43, RORgammat, or Foxp3-related pathways could further determine whether the predicted immune nodes are necessary for formula efficacy.

Third, metagenomics, metabolomics, transcriptomics, immune phenotyping, and formula-constituent profiling should be integrated to construct a ‘constituent-microbiota-metabolite-host target’ network. Only by moving from broad ‘multitarget regulation’ to testable chains such as specific constituent -> specific bacterial genus -> specific metabolite -> specific immune or endocrine outcome can the mechanistic depth and translational value of this field be improved.

## 6 Conclusion

Intervention in HT through the thyroid-gut axis using Chinese herbal formulas has theoretical plausibility and preliminary preclinical support. Current studies suggest that different formulas may cover different combinations of intestinal barrier, gut microbiota, immune homeostasis, and endocrine function. The four-level linkage model proposed in this review provides a clearer mechanistic framework for interpreting these findings and offers a stratified target-based approach for future experimental design. However, current evidence remains mainly derived from heterogeneous animal studies and is largely observational. High-quality clinical trials, causal microbiome experiments, standardized outcome systems, safety assessments, and pharmacokinetic studies are needed to move Chinese herbal formula intervention based on the thyroid-gut axis from theoretical hypothesis to evidence-based validation.
